# Goblint: Thread-Modular Abstract Interpretation Using Side-Effecting Constraints (Competition Contribution)

**DOI:** 10.1007/978-3-030-72013-1_28

**Published:** 2021-02-26

**Authors:** Simmo Saan, Michael Schwarz, Kalmer Apinis, Julian Erhard, Helmut Seidl, Ralf Vogler, Vesal Vojdani

**Affiliations:** 8 Eindhoven University of Technology, Eindhoven, The Netherlands; 9 Aalborg University, Aalborg East, Denmark; 10 University of Tartu, Tartu, Estonia; 11 Technische Universität München, Garching, Germany

## Abstract

Goblint is a static analysis framework for C programs specializing in data race analysis. It relies on thread-modular abstract interpretation where thread interferences are accounted for by means of flow-insensitive global invariants.

## 1 Verification Approach

Goblint is a static analyzer for C programs based on the framework of abstract interpretation [5]. It performs flow- and context-sensitive interprocedural analysis, using partial tabulation to handle procedure calls. The analysis of concurrent programs is thread-modular: analyzing each thread in isolation, as opposed to analyzing their interleavings. This scales well to larger programs with many threads. Interferences between threads happen through global variables, which are abstracted by a context- and flow-insensitive global invariant. When no other thread can interfere, copies of global variables are *privatized* within the local state. Their values may deviate from the global invariant due to local updates, thereby improving precision [11].

The analysis is specified using a side-effecting constraint system [3], in which right-hand sides of constraints can, during their evaluation, make additional contributions (*side effects*) to other constraint system variables. These side effects can be conveniently used both to express partial context-sensitivity of function calls and to add contributions to the global invariant. Such a constraint system is solved using a *local* generic solver, which yields a (post-)solution for just the reachable program points and contexts [1,8]. Solving is not strictly separated into widening and narrowing phases, but these may be intertwined instead [1]. Results of the analysis are reported only at the end based on the computed solution, as widening during the fixpoint computation might lead to spurious property violations, which later disappear due to narrowing.

⋆ Jury member

*Reachability Safety.* Reachability is mainly determined using value analysis, which, for integers, employs abstract domains based on intervals and exclusion sets. The value analysis also handles pointers (computing points-to information), heap memory (using allocation-site abstraction), structs, unions and arrays. The abstraction of arrays employs partitioning by the symbolic expression that is used to index into the array. On top of that, both global variables and heap-allocated memory are partitioned into disjoint regions [9].

*No Overflows.* The sound interval analysis is implemented using arbitrary precision integers. If the interval for an expression lies completely in the value range of its signed integer type, no overflow can occur at this location.

*No Data Race.* The main goal of Goblint is data race detection and its analyses have been optimized for this purpose. Mutexes may be handled both path-sensitively and symbolically. Memory accesses are partitioned (e.g., by heap region [9]), while locking expressions and access expressions are correlated using address equalities (e.g., a domain of affine and Herbrand equalities [10]) in order to analyze more sophisticated locking patterns [11].

## 2 Software Architecture

Goblint is implemented in OCaml and uses an updated fork of CIL [6] as its parser frontend for the C language. Since the latter requires preprocessed code, GCC is executed for preprocessing the input, although this step should be unnecessary on the SV-COMP benchmarks. No other major libraries or external tools are required.

The architecture of Goblint [2] is designed to be modular. Analyses, which are defined by their abstract domains and transfer functions, can be activated via runtime configuration options. A flexible query system allows for communication between analyses. Together, the combined analyses and the control-flow graphs of the functions in the program provide the side-effecting constraint system, which is solved by some local generic solver. While a number of solvers are available, the improved top-down solver TD3 [8] was employed for SV-COMP 2021. Post-processing the solution yields results for the analysis.

## 3 Strengths and Weaknesses

Due to over-approximation, abstract interpretation as employed by Goblint can only determine whether the correctness specification *must hold* or *may be violated*, but not whether a concrete violating execution exists. Therefore, to avoid a large number of false alarms due to imprecision in SV-COMP, Goblint only reports results "true" and "unknown" respectively. This is a clear limitation of our approach, as all competing tools do report definite violations. The strength of our approach, on the other hand, is that it aims to be sound by design (up to out-of-scope features of the input program as, e.g., inline assembler). This is evidenced by the fact that Goblint does not produce any incorrect results in the competition.

Goblint performs best in the *SoftwareSystems* and *ReachSafety-ProductLines* categories that consist of larger real-world programs, for which our approach is well suited. On the downside, our verifier performs poorly in reachability safety categories that contain smaller programs with intricate correctness conditions which our abstract domains cannot express.

Even though the support for checking overflows is very new in Goblint, it has some success in the *NoOverflows* category. Unfortunately, the tool has no success in *SoftwareSystems-*-NoOverflows*.

Although Goblint specializes in concurrency, it performs quite poorly in the *ConcurrencySafety* category. We believe this is because most benchmarks in the category require rather precise analysis of thread interleavings, which is not done in our thread-modular approach.

As Goblint has been optimized for data race detection, it unsurprisingly performs better in the *NoDataRace* demo category. It must be noted that the majority of benchmarks in the category were submitted from our own test suite, consisting of racy and race-free programs.

While the analyses can be fine-tuned via configuration options, the parameters are static and do not currently depend on the property nor the input program. A more granular and dynamic configuration system would allow increased precision, by enabling more expensive analyses where necessary, or decreased resource usage, by disabling unnecessary analyses, e.g., concurrency analyses on single-threaded programs. Furthermore, integrating counterexample-guided abstraction refinement (CEGAR) into our framework might allow Goblint to also report violations, while avoiding false alarms and gaining more precision.

## 4 Tool Setup and Configuration

Goblint version `svcomp21-0-g82e03b87` participated in SV-COMP 2021 [4,7]. It is available in both binary (Ubuntu 20.04) and source code form at our GitHub repository under the `svcomp21` tag.[3] The only runtime dependency is GCC. Instructions for building from source can be found in the `README`.

Both the tool-info module and the benchmark definition for SV-COMP are named `goblint`. They correspond to running the tool as follows:

```
./goblint --conf conf/svcomp21.json --sets ana.specification
property.prp input.c
```

Goblint participated in the following categories: *ReachSafety*, *ConcurrencySafety*, *NoOverflows*, *SoftwareSystems* (while opting-out from *SoftwareSystems-*-MemSafety*) and *NoDataRace* (demo category).

[3] https://github.com/goblint/analyzer/releases/tag/svcomp21

## 5 Software Project and Contributors

GOBLINT development takes place on GitHub,[4] while related publications are listed on its website.[5] It is an MIT-licensed joint project of the Technische Universität München (Chair of Formal Languages, Compiler Construction, Software Construction) and University of Tartu (Laboratory for Software Science).

*Acknowledgements.* This work was supported by Deutsche Forschungsgemeinschaft (DFG) – 378803395/2428 CONVEY and the Estonian Research Council grant PSG61. We would like to thank everyone who has contributed to GOBLINT over the years.
